# Consumption of Milk and alternatives decreased among Canadians from 2004 to 2015: evidence from the Canadian community health surveys

**DOI:** 10.1186/s40795-021-00465-9

**Published:** 2021-11-01

**Authors:** Hassan Vatanparast, Naorin Islam, Mojtaba Shafiee

**Affiliations:** 1 College of Pharmacy and Nutrition, University of Saskatchewan, 104 Clinic Place, Saskatoon, SK S7N 2Z4 Canada; 2 School of Public Health, University of Saskatchewan, Saskatoon, SK S7N 4Z2 Canada

**Keywords:** Milk & alternatives, Plain milk, Flavoured milk, Cheese, Yogurt, Canada’s food guide, Canadian population

## Abstract

**Background:**

Milk and milk products make important contributions to the diet of Canadians. The aim of this study was to examine trends in Milk & Alternatives consumption among Canadians (≥2 years) from 2004 to 2015.

**Methods:**

We used nutrition data from 2 nationally representative cross-sectional surveys conducted in 2004 and 2015 [Canadian Community Health Survey (CCHS) 2004 Cycle 2.2 and CCHS-Nutrition 2015] to compare Milk & Alternatives consumption between 2004 and 2015. Data from 24-h dietary recalls were collected using the Automated Multiple-Pass Method (AMPM).

**Result:**

From 2004 to 2015, the proportion of Canadians consuming Milk & Alternatives food group significantly decreased from 89.5 to 87.7% and the number of servings consumed per day dropped from 1.9 to 1.7. Despite their low energy contribution (12.3% of energy), Milk & Alternatives contributed 45.8% of calcium, 39.9% of vitamin D, and 36.0% of vitamin B12 to the diet of the Canadian population in 2015. Milk & Alternatives were among the top sources of vitamin A, phosphorus and riboflavin. Milk & Alternatives food group was a major contributor to saturated fat intake in both 2004 (31.2%) and 2015 (28.6%). In 2015, dietary intakes of calcium and vitamin D among Milk & Alternatives consumers were 137.8, and 59.4% higher, respectively, than those of non-consumers.

**Conclusion:**

Daily intake of Milk & Alternatives has decreased in the Canadian population over time, which may adversely affect the nutritional profile of the diet.

## Background

Milk and dairy products make important contributions to the diet of Canadians and provide significant amounts of protein, vitamin A, vitamin D, vitamin B12, and riboflavin as well as calcium, phosphorus, magnesium, and potassium [1]. Due to providing a wide range of nutrients, milk and dairy products have been a part of the Canada’s Food Guides since the 1940s [2]. The 2007 Canada’s Food Guide to Healthy Eating provided specific recommendations for four food groups, including: Grain Products, Vegetables and Fruit, Milk and Alternatives, and Meat and Alternatives [3]. However, the new Canada’s Food Guide (2019) has eliminated the “Milk & Alternatives” food group and recommended lower-fat dairy products as a subgroup of protein foods [4]. This major change in the new Canada’s Food Guide may affect the intake of milk and dairy products in the Canadian population.

It has been shown that nutrients from dairy foods are difficult to replace, and their replacement with calcium-equivalent foods may adversely affect the nutritional profile of the diet [1]. Thus, information concerning trends in consumption of milk and alternatives is essential to describe the patterns of intake and to prevent potential nutritional deficiencies. Recent evidence suggests a decreasing trend in the consumption of dairy products in developed countries such as the USA, Finland and Britain [5–7]. A limited number of studies have evaluated trends in consumption of milk and alternatives in the Canadian population over time [8–11]. Using nationally representative data from Canadian Community Health Surveys, Tugault-Lafleur et al. showed that Canadians reported consuming significantly fewer servings of fluid milk while consuming significantly more dairy products (i.e., cheese, yogurt) from 2004 to 2015 [8]. Jones et al. also reported that compared with 2004, per capita daily consumption of plain milk for the Canadian population decreased by 35% for energy (− 35.3 kcal) and 37% for volume (− 76.0 ml) in 2015 [9]. Moreover, it has been found that from 2004 to 2015, adjusted sales volume increased for novel categories of sugary drinks, such as drinkable yoghurt and flavoured milk [10]. We have previously reported a decreasing trend in the consumption of plain milk and an increasing trend in the consumption of plant-based beverages from 2004 to 2015 [12]. However, to date, no study has determined and compared the percentage of Canadians consuming milk and alternatives, the contribution of milk and alternatives to daily energy and nutrient intake, and the prevalence of meeting recommendations for this food group in the Canadian population between 2004 and 2015.

Using nutrition data from 2 nationally representative surveys conducted in 2004 and 2015, our main objective was to examine changes in the proportion of Canadians consuming Milk & Alternatives food group and its major food items (i.e., plain milk, flavoured milk, cheese, yogurt and fortified soy beverages), and to examine changes in the number of daily servings of Milk & Alternatives. We also aimed to 1) determine and compare the prevalence of meeting recommendations for Milk & Alternatives food group among Milk & Alternatives consumers between 2004 and 2015, 2) determine and compare the percent contribution of Milk & Alternatives food group and its major food items to daily energy and nutrient intakes in Milk & Alternatives consumers between 2004 and 2015, and 3) determine daily energy and nutrient intakes of Milk & Alternatives food group consumers and non-consumers in 2004 and 2015.

## Methods

### Study design, data source, and dietary data collection

This study was based on the 2004 and 2015 Canadian Community Health Survey (CCHS) data of Statistics Canada, which are cross-sectional surveys. The data was collected using the Automated Multiple Pass Method (AMPM) [13]. The survey data was collected at two time points - day 1 and day 2 for both survey years. The information was collected from January 2015 to December 2015. The response rate of these surveys was 76.5% in 2004 and 62% in 2015. The data was obtained from 35,107 (CCHS 2004) and 20,487 (CCHS 2015) respondents who were 2 year and over, residing in ten provinces of Canada at day 1. The survey respondents who are aged 1 to 5 years old participated in the survey using proxy interview by their parents or guardians. The survey was conducted for children 6–11 years with guidance of their parents or guardian. Individuals 12 years and over were interviewed and responded by themselves.

The analysis was performed at the Saskatchewan Research Data Centre of Statistics Canada after getting approval from Statistics Canada. The data includes the dietary/nutritional information of Canadians using 24-h recall. This survey data also includes socio-demographic variables of Canadians such as sex, age, ethnicity, marital status, food security, Body mass index (BMI), obesity, area of residence, income, education, smoking status, immigration status. The variables in this study were measured based on CCHS survey protocols. The details of measurements are provided in Statistics Canada website [14, 15]. All procedures involving research study participants were approved by Statistics Canada.

### Analytical sample

This study was restricted to all Canadians 2 years and over except pregnant or lactating women and subjects without any reported food items, as describe elsewhere [12]. Extreme positive outliers were excluded from the study as those dietary intakes represented very high unrealistic nutrient intake. For this study, we included the dietary intake at day 1. The final sample size of this study was 29,721,941 in 2004 and 33,513,207 in 2015 (weighted frequency) after excluding children below 2 years, pregnant/lactating women, extreme positive five nutrient intake of each nutrient and persons who did not report any food consumption information. We divided the population by age group (2–5 years, 6–12 years, 13–18 years, 19–54 years and ≥ 55 years).

### Milk & alternatives food group and the main food items

This study included Milk & Alternatives food group as one of the four main food groups in 2007 Canada’s Food Guide at the time of the survey. This food group includes plain milk, flavoured milk, cheese, yogurt, fortified soy beverages, and other food items as indicated in appendix. In this study, we only included “Milk & Alternatives” as the main food group and plain milk, flavoured milk, cheese, yogurt, and fortified soy beverages as the main food items. The consumption of the “Milk & Alternatives” food group and the main food items were compared between 2004 and 2015. We defined Milk & Alternatives consumers as those who reported consuming any of the food items in Milk & Alternatives food group on day one 24-h recall. The socio-demographic characteristics were compared between children (2–18 years) and adult (≥ 19 years) Milk & Alternatives consumers and non-consumers in 2004 and 2015. It should be noted that changes to the food booklet used in 2004 and 2015 have resulted in some portion size changes, especially in the case of beverages. Line drawings of the glasses, plates and bowls in 2004 were replaced with actual-size photographs in 2015 that gave a more realistic three-dimensional view of the items [8]. We also calculated the percentages of individuals meeting the minimum dietary recommended serving size of Milk & Alternatives per day. The recommended serving sizes of Milk & Alternatives was based on the 2007 Canada’s Food Guide which was in practice at the time of the survey [3].

### Daily nutrients intake

The contribution of Milk & Alternatives to daily nutrient intakes were assessed in the total population (≥2 years) and each of the five age groups (2–5 years, 6–12 years, 13–18 years, 19–54 years, ≥55 years) and compared between 2004 and 2015. We also calculated the nutrient contribution of the plain milk, flavoured milk, cheese, yogurt and fortified soy beverages to daily intake for the total population (≥2 years). The daily nutrient intakes of Milk & Alternatives consumers and non-consumers was compared between 2004 and 2015.

### Serving sizes

The serving sizes of Milk & Alternatives food group and main food items (plain milk, flavoured milk, cheese, yogurt, fortified soy beverages) were calculated in this study. The serving sizes of the “Milk & Alternatives” group depend on the milk products, and for the food items, the serving sizes were 250 mL/1 cup for plain milk, flavoured milk and fortified soy beverages, 50 g for cheese, and 175 g for yogurt. The serving sizes were compared between 2004 and 2015. The results using serving sizes were presented in five age groups for Milk & Alternatives, plain milk, flavoured milk, cheese and yogurt. We were not able to release data about fortified soy beverages intake across five age groups because of small cell frequency (less than five) which was the violation of vetting protocol of Saskatchewan Research Data Centre [16].

### Sociodemographic variables

The socio-demographic variables included in this study were age, sex (male, female), ethnicity (white, non-white), marital status (married, not married), education (university graduate: if any individual in a household is a university graduate, not university graduate: if any individual in a household is not a university graduate), area of residence (urban, rural), and immigration status (yes, no). We also used the Household Food Security Survey Module (HFSSM) to measure food insecurity over the past 12 months (yes, no) [14, 15]. It was defined as “yes” if the respondent reported to be food secure and “no” if the respondent reported marginal, moderate or severe food insecurity. Smoking status was defined as “yes” if the individual was current smoker and “no” if the individual was not current smoker. Similarly, immigration status was defined as “yes” if the participant was immigrant to Canada and “no” if the participant was not immigrant to Canada.

### Anthropometric variables

BMI was calculated as weight (kg) divided by height squared (m^2^). Other anthropometric variables included in this analysis were BMI z-score, children obesity (yes, no), and adult obesity (yes, no). Child obesity was defined as “yes” if the child was either overweight or obese and as “no” if the child was not overweight or obese. We applied the same definition for adult obesity.

### Statistical analyses

The statistical analyses were performed using SAS (version 9.4, SAS Institute) at the Saskatchewan Research Data Centre of Statistics Canada. The results were adjusted using appropriate bootstrapping weights to produce population-level estimates [17]. The values are presented as mean ± SE or percentages ± SE. The chi-square test was performed for the comparison of sociodemographic variables between Milk & Alternatives consumers and non-consumers. In all the other analyses, tables and figures, we compared the results between 2004 and 2015 using the “overlap of confidence interval” approach [18]. We also compared then serving sizes of milk and alternatives and prevalence of meeting recommendations for Milk & Alternatives food group using the same approach. The percent contribution of Milk & Alternatives food group to daily energy and nutrient intakes in Milk & Alternatives consumers were evaluated by ANCOVA, where we adjusted the model by daily energy intake and compared the results between 2004 and 2015. The same model was applied for the percent nutrient contribution of plain milk, flavoured milk, cheese, yogurt and fortified soy beverages to daily intake to compare the results between 2004 and 2015. The comparison of the mean daily nutrient intake of “Milk & Alternatives” between consumers and non-consumers were performed using ANCOVA (adjusted by daily energy intake). The statistical analyses were performed at 5% level of significance in this study i.e. all the significant outputs stated in the result section are statistically significant (*p*-value< 0.05) at 5% level of significance.

## Results

Sociodemographic characteristics of Canadian children and adults as milk & alternatives consumers and non-consumers are presented in Table 1. Among children and adolescents (2–18 years), Milk & Alternatives consumers were younger than Milk & Alternatives non-consumers (*p*-value< 0.05) in both 2004 (10.3 years vs 12.6 years, *p* < 0.05) and 2015 (9.7 years vs 12.5 years, p < 0.05). The percentage of White was higher (*p*-value< 0.05) in Milk & Alternatives consumers than in Milk & Alternatives non-consumers in 2004. In both 2004 and 2015, the percentage of food secured children were significantly (p-value< 0.05) higher among consumers compared to non-consumers. The percentage of immigrants to Canada was higher (*p*-value< 0.05) in Milk & Alternatives non-consumers compared to consumers in both 2004 and 2015.

**Table 1:** Sociodemographic characteristics of Canadian children (2–18 years) and adults (≥19 years) as Milk & Alternatives consumers and non-consumers^1^

Characteristic	CCHS 2004		CCHS 2015		CCHS 2004		CCHS 2015	
	Children/teens (2–18 years)		Children/teens (2–18 years)		Adults (≥19 years)		Adults (≥19 years)	
	Consumers [***n*** = 6,120,154 (94.2%^**3**^)]	Non-consumers [***n*** = 378,400 (5.8%)]	Consumers [***n*** = 5,993,085 (92.9%)]	Non-consumers [***n*** = 454,809 (7.1%)]	Consumers [***n*** = 20,499,396 (88.3%)]	Non-consumers [***n*** = 2,723,991 (11.7%)]	Consumers [***n*** = 23,387,890 (86.4%)]	Non-consumers [***n*** = 3,677,423 (13.6%)]
Age (y)	10.3 ± 0.03	12.6 ± 0.2*	9.7 ± 0.1	12.5 ± 0.4*	46.6 ± 0.1	45.5 ± 0.6	49.5 ± 0.2	47.9 ± 0.7*
Sex (% male)	51.3 ± 0.5	50.4 ± 3.2	50.2 ± 0.9	50.4 ± 4.0	49.2 ± 0.3	56.1 ± 2.0*	45.8 ± 2.3	54.2 ± 2.3*
Ethnicity (% White)	82.4 ± 0.6	72.5 ± 3.1*	68.1 ± 1.4	59.5 ± 4.5	86.0 ± 0.6	73.1 ± 2.0*	77.0 ± 1.0	62.3 ± 2.6
Education (% university grad)	30.9 ± 0.7	30.7 ± 3.3	44.4 ± 1.2	46.3 ± 4.2	30.5 ± 0.7	29.0 ± 2.0	38.7 ± 1.0	36.8 ± 2.6
Smoker (% yes)	11.0 ± 0.7	13.9 ± 2.4	3.8 ± 0.5	3.1 ± 1.3	24.2 ± 0.6	32.0 ± 1.9*	9.4 ± 2.7	19.1 ± 0.7*
Marital status (% married or co-habiting)	n/a	n/a	n/a	n/a	65.1 ± 0.7	59.0 ± 1.9*	64.2 ± 0.9	63.1 ± 2.4
Food secure (% yes)	84.2 ± 0.5	79.5 ± 2.5*	84.3 ± 0.8	80.2 ± 3.6	88.6 ± 0.5	82.7 ± 1.5*	89.1 ± 0.6	85.3 ± 1.7*
BMI (kg/m^2^)	n/a	n/a	n/a	n/a	27.1 ± 0.1	27.2 ± 0.3	27.3 ± 0.1	27.1 ± 0.3
BMI z-score^2^	0.6 ± 0.02	0.5 ± 0.1	0.44 ± 0.0	0.39 ± 0.2	n/a	n/a	n/a	n/a
Overweight/obese (% yes)	26.4 ± 0.8	25.3 ± 3.5	25.8 ± 1.1	30.5 ± 4.6	59.7 ± 0.9	63.4 ± 2.9	62.2 ± 1.1	59.3 ± 2.8
Urban residence (% yes)	81.1 ± 0.7	82.3 ± 2.7	82.2 ± 1.0	81.6 ± 3.5	82.0 ± 0.7	85.6 ± 1.3*	81.9 ± 0.8	86.5 ± 1.6*
Immigrant to Canada (% yes)	6.3 ± 0.4	10.7 ± 1.9*	8.6 ± 0.6	15.1 ± 2.9*	22.1 ± 0.6	34.9 ± 2.1*	25.7 ± 1.0	38.3 ± 2.6*

^1^All data are weighted and bootstrapped to obtain population level estimate. * Significant differences between consumer and non-consumers at 5% level of significance. ^2^The BMI z-score for children 5-18 years. ^3^The percentages of Milk & Alternatives consumers and non-consumers in CCHS 2004 and 2015. Values are presented as mean ± SE or % ± SE. CCHS: Canadian Community Health Survey; BMI: Body Mass Index.

Among Canadian adults (> 19 years), Milk & Alternatives consumers were older than Milk & Alternatives non-consumers in 2015 (49.5 years vs 47.9 years, *p* < 0.05). A lower percentage (*p*-value< 0.05) of males was observed in Milk & Alternatives consumers compared to non-consumers in both 2004 and 2015. The percentage of White was higher (*p*-value< 0.05) in Milk & Alternatives consumers than in Milk & Alternatives non-consumers in 2004. In both 2004 and 2015, Milk & Alternatives non-consumers were more likely (*p*-value< 0.05) to be current smokers, food insecure, urban residence and immigrants than Milk & Alternatives consumers.

### Daily nutrient intakes of Milk & alternatives food group consumers and non-consumers

As reported in Table 2, the amount of total energy intake among Milk & Alternatives consumers were 22.3 and 24.0% higher, than those of non-consumers in 2004 and 2015, respectively. In 2004, Milk & Alternatives consumers obtained 158.5% more calcium, 93.5% more vitamin D and 67.9% more saturated fat than Milk & Alternatives non-consumers. In 2015, dietary intakes of calcium, vitamin D and saturated fat among Milk & Alternatives consumers were 137.8, 59.4 and 55.6% higher, respectively than those of non-consumers.

**Table 2:** Daily energy and nutrient intakes of Milk & Alternatives food group consumers and non-consumers in 2004 and 2015

	Daily nutrient intakes					
	CCHS 2004			CCHS 2015		
	Milk & alternatives consumers (***n*** = 26,619,550)	Milk & alternatives non-consumers (***n*** = 3,102,391)	Percent difference	Milk & alternatives consumers (***n*** = 29,380,975)	Milk & alternatives non-consumers (***n*** = 4,132,232)	Percent difference
**Calcium (mg)**	968.2 ± 6.7	374.5 ± 8.7*	158.5% ^1^	873.5 ± 7.4 ¥	367.3 ± 10.6*	137.8% ^1^
**Vitamin D (**μg**)**	6.0 ± 0.1	3.1 ± 0.2*	93.5% ^2^	5.1 ± 0.1 ¥	3.2 ± 0.3*	59.4% ^2^
**Saturated Fat (g)**	26.2 ± 0.2	15.6 ± 0.3*	67.9% ^3^	23.8 ± 0.2 ¥	15.3 ± 0.7*	55.6% ^3^
**Vitamin A (**μg**)**	699.8 ± 8.2	428.2 ± 17.5*	63.4% ^4^	655.4 ± 8.5 ¥	477.6 ± 30.2*	37.2%
**Riboflavin (mg)**	2.04 ± 0.01	1.28 ± 0.02*	59.4% ^5^	1.96 ± 0.01 ¥	1.32 ± 0.03*	48.5% ^4^
**Phosphorus (mg)**	1396 ± 8	918 ± 17*	52.1%	1315 ± 9 ¥	950 ± 27*	38.4% ^5^
**Total Sugar (g)**	113.5 ± 0.8	79.1 ± 2.4*	43.5%	93.7 ± 0.8 ¥	69.7 ± 2.6	34.4%
**Total Fat (g)**	77.9 ± 0.6	58.8 ± 1.2*	32.5%	70.6 ± 0.6 ¥	56.1 ± 2.2*	25.8%
**Thiamin (mg)**	1.77 ± 0.01	1.35 ± 0.7*	31.1%	1.61 ± 0.01 ¥	1.26 ± 0.03*	27.8%
**Sodium (mg)**	3165 ± 23	2499 ± 52*	26.7%	2772 ± 23 ¥	2150 ± 63*	28.9%
**Vitamin B12 (μg)**	4.3 ± 0.1	3.4 ± 0.2*	26.5%	4.1 ± 0.1 ¥	3.1 ± 0.2*	32.3%
**Potassium (mg)**	3119 ± 16	2474 ± 44*	26.1%	2682 ± 17 ¥	2233 ± 55*	20.1%
**Magnesium (mg)**	324.8 ± 1.7	258.1 ± 5.0*	25.8%	302.4 ± 2.2 ¥	263.7 ± 7.8*	14.7%
**MUFA (g)**	30.9 ± 0.2	24.8 ± 0.5*	24.6%	26.0 ± 0.3 ¥	22.6 ± 1.0	15.0%
**Energy (kcal)**	2140 ± 11	1750 ± 29	22.3%	1910 ± 11.8 ¥	1540 ± 41	24.0%
**Iron (mg)**	14.4 ± 0.1	11.8 ± 0.2*	22.0%	12.5 ± 0.1 ¥	10.3 ± 0.3*	21.4%
**Folate (mg)**	464.6 ± 3.2	381.5 ± 8.6*	21.8%	450.4 ± 3.6 ¥	348.0 ± 8.9	29.4%
**Carbohydrates (g)**	269.1 ± 1.5	221.9 ± 4.6*	21.3%	232.1 ± 1.5 ¥	186.2 ± 4.1*	24.7%
**Zinc (mg)**	11.3 ± 0.1	9.4 ± 0.2*	20.2%	10.5 ± 0.1 ¥	8.6 ± 0.3*	22.1%
**Protein (g)**	85.5 ± 0.6	72.1 ± 1.5*	18.6%	79.2 ± 0.5 ¥	66.3 ± 2.1*	19.5%
**Vitamin C (mg)**	133.4 ± 1.4	113.2 ± 4.0*	17.8%	102.4 ± 1.5 ¥	91.2 ± 4.8	12.3%
**Niacin (mg)**	39.2 ± 0.3	35.0 ± 0.6*	12.0%	38.5 ± 0.3	35.3 ± 1.2*	9.1%
**Vitamin B6 (mg)**	1.85 ± 0.01	1.66 ± 0.03*	11.4%	1.63 ± 0.01 ¥	1.57 ± 0.1	3.8%
**PUFA (g)**	13.3 ± 0.1	12.2 ± 0.3*	9.0%	14.5 ± 0.2 ¥	13.3 ± 0.5	9.0%
**Cholesterol (mg)**	273.1 ± 3.2	252.1 ± 8.3	8.3%	265.1 ± 3.4	221.5 ± 11.6*	19.7%

^1^All data are weighted and bootstrapped to obtain population level estimate. * Significant differences between 2004 and 2015 at 5% level of significance. Values are presented as mean ± SE

### Percent of Canadians consuming Milk & alternatives and number of servings of Milk & alternatives per day

As reported in Table 3, the percentage of individuals consuming Milk & Alternatives significantly decreased from 89.5% in 2004 to 87.7% in 2015. However, this decrease was not significant when stratified across age groups. In 2015, the highest daily Milk & Alternatives consumption was observed in children aged 2–5 years (97.6%) and the lowest in adults aged 19–54 years (85.8%). From 2004 to 2015, the percentage of individuals consuming plain milk significantly decreased from 70.2 to 56.1% (*p* < 0.05). Children aged 6–12 years and adults aged ≥55 years exhibited a significant rise in the percentage of flavoured milk consumption (from 7.5 to 11.1% and from 0.7 to 1.8%, respectively). From 2004 to 2015, no statistically significant change was observed in the percentage of individuals consuming cheese. The percentage of individuals consuming yogurt significantly increased from 14.2% in 2004 to 20.4% in 2015 (*p* < 0.05). The percentage of Canadians consuming fortified soy beverages also significantly increased from 0.50% in 2004 to 1.51% in 2015.

**Table 3:** Percent of Canadians consuming Milk & Alternatives and number of servings per day by age groups^1^

		Milk & Alternatives food group		Main Food Items									
				Plain milk		Flavoured milk		Cheese		Yogurt		Fortified soy beverages	
		CCHS 2004 (***n*** = 26,619,550)	CCHS 2015 (***n*** = 29,380,975)	CCHS 2004 (***n*** = 20,879,908)	CCHS 2015 (***n*** = 18,790,221)	CCHS 2004 (***n*** = 890,305)	CCHS 2015 (***n*** = 1,118,649)	CCHS 2004 (***n*** = 16,392,316)	CCHS 2015 (***n*** = 17,926,472)	CCHS 2004 (***n*** = 4,217,387)	CCHS 2015 (***n*** = 6,827,004)	CCHS 2004 (***n*** = 146,085)	CCHS 2015 (***n*** = 506,575)
Percent of consumption (%)	**All ages**	89.5 ± 0.4	87.7 ± 0.5*	70.2 ± 0.5	56.1 ± 0.7*	3.0 ± 0.2	3.3 ± 0.2	55.1 ± 0.6	53.5 ± 0.8	14.2 ± 0.4	20.4 ± 0.6*	0.50 ± 0.11	1.51 ± 1.15*
	**Age groups** 2–5 years	97.9 ± 0.5	97.6 ± 0.6	87.4 ± 1.1	81.3 ± 2.0	7.6 ± 0.9	7.5 ± 1.3	59.8 ± 1.6	66.5 ± 2.2	25.7 ± 1.5	47.7 ± 2.5*	–	–
	6–12 years	95.8 ± 0.4	95.1 ± 0.8	81.4 ± 0.9	71.2 ± 1.6*	7.5 ± 0.6	11.1 ± 1.2*	64.1 ± 1.0	60.4 ± 1.7	17.3 ± 0.8	29.6 ± 1.6*	–	–
	13–18 years	91.4 ± 0.7	88.6 ± 1.1	70.0 ± 1.0	57.9 ± 1.4*	6.3 ± 0.5	8.0 ± 0.8	62.6 ± 1.0	58.2 ± 1.4	11.4 ± 0.7	14.0 ± 1.0	–	–
	19–54 years	88.1 ± 0.6	85.8 ± 0.9	66.0 ± 0.8	50.0 ± 1.2*	2.4 ± 0.3	2.1 ± 0.3	56.6 ± 0.9	53.8 ± 1.3	14.4 ± 0.7	18.6 ± 1.0*	–	–
	≥ 55 years	88.6 ± 0.6	87.3 ± 0.8	73.2 ± 0.8	58.3 ± 1.2*	0.7 ± 0.1	1.8 ± 0.3*	45.4 ± 1.0	48.7 ± 1.2	12.2 ± 0.6	19.3 ± 1.0*	–	–
Servings per day (n/day)	**Total**	1.9 ± 0.02	1.7 ± 0.02*	1.2 ± 0.01	0.9 ± 0.01*	1.5 ± 0.05	1.3 ± 0.05*	0.7 ± 0.01	0.8 ± 0.01*	0.8 ± 0.03	0.8 ± 0.02	0.9 ± 0.09	0.8 ± 0.06
	**Age groups** 2–5 years	2.5 ± 0.05	2.4 ± 0.08	1.7 ± 0.04	1.5 ± 0.05	1.1 ± 0.14	0.9 ± 0.08	0.5 ± 0.03	0.6 ± 0.04	0.6 ± 0.03	0.8 ± 0.05*	–	–
	6–12 years	2.5 ± 0.04	2.2 ± 0.04*	1.5 ± 0.03	1.3 ± 0.03*	1.2 ± 0.05	0.9 ± 0.03*	0.6 ± 0.02	0.7 ± 0.02	0.7 ± 0.03	0.8 ± 0.04*	–	–
	13–18 years	2.5 ± 0.04	2.2 ± 0.05*	1.7 ± 0.03	1.4 ± 0.04*	1.7 ± 0.10	1.5 ± 0.12	0.7 ± 0.02	0.9 ± 0.03*	0.8 ± 0.04	0.8 ± 0.04	–	–
	19–54 years	1.8 ± 0.03	1.6 ± 0.03*	1.1 ± 0.02	0.8 ± 0.03*	1.6 ± 0.09	1.7 ± 0.11	0.7 ± 0.02	0.8 ± 0.03*	0.9 ± 0.06	0.8 ± 0.03	–	–
	≥ 55 years	1.5 ± 0.03	1.5 ± 0.03	0.9 ± 0.02	0.8 ± 0.02*	1.3 ± 0.17	1.2 ± 0.13	0.6 ± 0.02	0.7 ± 0.02*	0.8 ± 0.02	0.8 ± 0.03	–	–

^1^All data are weighted and bootstrapped to obtain population level estimate. * Significant differences between 2004 and 2015 at 5% level of significance. Values are presented as % ± SE. CCHS: Canadian Community Health Survey.

Among Milk & Alternatives consumers (≥ 2 years), the mean number of servings of Milk & Alternatives per day significantly decreased from 1.9 servings in 2004 to 1.7 servings in 2015 (Table 3). The mean daily number of servings of Milk & Alternatives significantly decreased in all age groups except children aged 2–5 years and adults aged ≥55 years. In 2015, the highest mean number of servings of Milk & Alternatives was found in children aged 2–5 years (2.4 servings/day) and the lowest in adults aged ≥55 years (1.5 servings/day). From 2004 to 2015, the mean daily number of servings of plain milk significantly decreased from 1.2 servings to 0.9 servings. The mean daily number of servings of cheese significantly increased from 0.7 servings in 2004 to 0.8 servings in 2015. Overall, no statistically significant change was observed in the mean daily number of servings of yogurt between 2004 and 2015. The mean daily number of servings of fortified soy beverages remained statistically unchanged between 2004 and 2015.

### Prevalence of meeting recommendations for Milk & alternatives food group

**Fig. 1:**
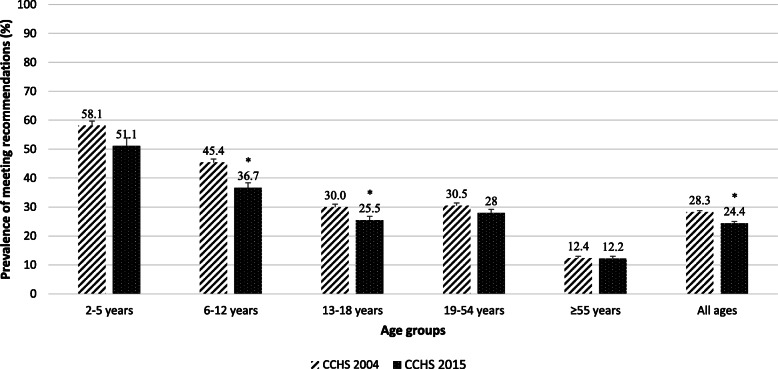
Prevalence of meeting recommendations for Milk & Alternatives food group among Milk & Alternatives consumers in 2004 (*n* = 26,619,550) and 2015 (*n* = 29,380,975). All data are weighted and bootstrapped to obtain population level estimate. Recommended number of servings: 2–8 years: 2 servings/day; 9–13 years: 3–4 servings/day; 14–18 years: 3–4 servings/day; 19–50 years: 2 servings/day; and ≥ 51 years: 3 servings/day. * Significant differences between 2004 and 2015 at 5% level of significance

As shown in Fig. 1, the prevalence of meeting recommendations for Milk & Alternatives food group among Canadian Milk & Alternatives consumers significantly decreased from 28.3% in 2004 to 24.4% in 2015. From 2004 to 2015, the prevalence of meeting recommendations for Milk & Alternatives food group significantly decreased in children aged 6–12 years (from 45.4 to 36.7%) and adolescents aged 13–18 years (from 30.0 to 25.5%). In both 2004 and 2015, children aged 2–5 years exhibited the highest prevalence of meeting recommendations (58.1 and 51.1%, respectively) and adults aged ≥55 years exhibited the lowest prevalence of meeting recommendations (12.4 and 12.2%, respectively) for Milk & Alternatives food group.

### Percent contributions of Milk & alternatives to daily nutrient intakes

Among Milk & Alternatives consumers (≥ 2 years), the percent contribution of energy from the Milk & Alternatives food group significantly decreased from 13.2% in 2004 to 12.3% in 2015 (Table 4). In 2004, Milk & Alternatives contributed substantially to daily intake of calcium (51.9%), vitamin D (46.2%), vitamin B12 (38.8%), vitamin A (35.1%), saturated fat (31.2%), phosphorus (30.8%), riboflavin (28.2%), cholesterol (24.5%), protein (21.3%) and zinc (20.1%). Similarly, in 2015, Milk & Alternatives provided over 40% of the daily calcium intake, over 30% of vitamin D, vitamin B12, and vitamin A, and over 20% of saturated fat, phosphorus, riboflavin, and cholesterol.

**Table 4:** Percent contribution of Milk & Alternatives food group to daily energy and nutrient intakes in Milk & Alternatives consumers in 2004 and 2015^#^

	Total population		2–5 years		6–12 years		13–18 years		19–54 years		≥ 55 years	
	CCHS 2004 (***n*** = 26,619,550)	CCHS 2015 (***n*** = 29,380,975)	CCHS 2004 (***n*** = 921,308)	CCHS 2015 (***n*** = 1,198,275)	CCHS 2004 (***n*** = 2,557,706)	CCHS 2015 (***n*** = 2,467,181)	CCHS 2004 (***n*** = 2,641,141)	CCHS 2015 (***n*** = 2,327,629)	CCHS 2004 (***n*** = 13,867,241)	CCHS 2015 (***n*** = 13,948,151)	CCHS 2004 (***n*** = 6,632,155)	CCHS 2015 (***n*** = 9,439,739)
Energy (%)	**13.2 ± 0.1**	12.3 ± 0.2 *	**23.1 ± 0.5**	**23.8 ± 0.7**	**17.3 ± 0.3**	**16.9 ± 0.4**	**15.2 ± 0.2**	**14.5 ± 0.3**	**12.2 ± 0.2**	11.0 ± 0.3 *	**11.5 ± 0.2**	**11.1 ± 0.2**
Calcium (%)	51.9 ± 0.2 ^1^	45.8 ± 0.4 ^1^*	67.0 ± 0.7 ^2^	65.6 ± 0.9 ^1^	61.7 ± 0.5 ^1^	56.8 ± 0.7 ^1^*	58.8 ± 0.5 ^1^	52.9 ± 0.7 ^1^*	50.1 ± 0.4 ^1^	43.4 ± 0.7 ^1^*	47.0 ± 0.4 ^1^	42.2 ± 0.6 ^1^*
Vitamin D (%)	46.2 ± 0.3 ^2^	39.9 ± 0.5 ^2^*	67.9 ± 0.9 ^1^	62.7 ± 1.7 ^2^*	60.9 ± 0.7 ^2^	56.2 ± 1.0 ^2^*	56.0 ± 0.7 ^2^	46.9 ± 1.0 ^2^*	42.4 ± 0.6 ^2^	36.4 ± 0.9 ^2^*	41.4 ± 0.6 ^2^	36.1 ± 0.7 ^2^*
Vitamin B12 (%)	38.8 ± 0.3 ^3^	36.0 ± 0.4 ^3^*	59.3 ± 0.9 ^3^	58.9 ± 1.4 ^3^	50.3 ± 0.6 ^3^	50.5 ± 1.0 ^3^	45.3 ± 0.6 ^4^	43.3 ± 0.9 ^4^	36.2 ± 0.5 ^3^	32.0 ± 0.7 ^3^*	34.2 ± 0.5 ^3^	33.4 ± 0.5 ^3^
Vitamin A (%)	35.1 ± 0.3 ^4^	33.8 ± 0.4 ^4^	50.3 ± 0.9 ^4^	50.9 ± 1.4 ^4^	46.1 ± 0.6 ^4^	45.2 ± 0.9 ^4^	46.5 ± 0.6 ^3^	43.6 ± 0.9 ^3^*	33.0 ± 0.4 ^4^	31.4 ± 0.7 ^4^	28.5 ± 0.4 ^4^	29.8 ± 0.6 ^4^
Saturated Fat (%)	31.2 ± 0.3 ^5^	28.6 ± 0.3 ^5^*	47.9 ± 0.7 ^6^	47.7 ± 1.0 ^5^	38.5 ± 0.5 ^6^	36.8 ± 0.7 ^6^	35.1 ± 0.5 ^6^	32.4 ± 0.7 ^5^*	30.1 ± 0.4 ^5^	26.9 ± 0.6 ^5^*	27.0 ± 0.4 ^5^	25.7 ± 0.5 ^5^
Phosphorus (%)	30.8 ± 0.2 ^6^	25.4 ± 0.3 ^6^*	48.3 ± 0.6 ^5^	44.2 ± 0.9 ^7^*	41.2 ± 0.4 ^5^	35.0 ± 0.6 ^7^*	37.3 ± 0.4 ^5^	30.6 ± 0.6 ^7^*	28.8 ± 0.3 ^6^	22.9 ± 0.4 ^6^*	26.1 ± 0.3 ^6^	22.9 ± 0.4 ^6^*
Riboflavin (%)	28.2 ± 0.2 ^7^	25.0 ± 0.3 ^7^*	45.7 ± 0.7 ^7^	47.4 ± 1.0 ^6^	37.5 ± 0.4 ^7^	37.8 ± 0.7 ^5^	33.8 ± 0.4 ^7^	31.7 ± 0.6 ^6^*	25.9 ± 0.3 ^7^	21.3 ± 0.5 ^7^*	24.9 ± 0.3 ^7^	22.6 ± 0.4 ^7^*
Cholesterol (%)	24.5 ± 0.3 ^8^	23.0 ± 0.4 ^8^*	40.1 ± 0.9 ^8^	39.3 ± 1.2 ^8^	33.8 ± 0.6 ^8^	30.9 ± 0.7 ^8^*	30.1 ± 0.5 ^8^	27.7 ± 0.8 ^8^	23.0 ± 0.5 ^8^	21.3 ± 0.6 ^8^	19.8 ± 0.4 ^8^	20.1 ± 0.5 ^8^
Protein (%)	21.3 ± 0.2 ^9^	18.7 ± 0.2 ^9^*	36.0 ± 0.6 ^9^	34.7 ± 0.8 ^9^	29.4 ± 0.4 ^9^	26.2 ± 0.5 ^10^*	25.9 ± 0.4 ^9^	22.7 ± 0.5 ^10^*	19.7 ± 0.3 ^9^	16.7 ± 0.4 ^9^*	17.9 ± 0.3 ^9^	16.8 ± 0.3 ^9^
Zinc (%)	20.1 ± 0.2 ^10^	18.6 ± 0.2 ^10^*	33.5 ± 0.6 ^10^	34.6 ± 0.8 ^10^	27.2 ± 0.4 ^10^	26.5 ± 0.5 ^9^	24.1 ± 0.3 ^10^	23.2 ± 0.5 ^9^	18.7 ± 0.3 ^10^	16.6 ± 0.4 ^10^*	16.6 ± 0.3 ^10^	16.4 ± 0.3 ^10^
Total Fat (%)	18.3 ± 0.2	17.3 ± 0.2	32.1 ± 0.6	31.7 ± 0.9	23.8 ± 0.4	23.1 ± 0.5	20.8 ± 0.3	19.8 ± 0.5	17.3 ± 0.3	15.9 ± 0.4 *	15.5 ± 0.3	15.3 ± 0.4
Total Sugar (%)	18.1 ± 0.2	17.0 ± 0.3 *	27.0 ± 0.6	31.2 ± 1.0 *	21.9 ± 0.4	22.8 ± 0.6	19.9 ± 0.4	19.3 ± 0.6	17.1 ± 0.4	14.7 ± 0.4 *	16.9 ± 0.3	16.5 ± 0.4
Potassium (%)	16.9 ± 0.1	14.0 ± 0.2 *	31.8 ± 0.6	30.1 ± 0.9	26.1 ± 0.4	22.8 ± 0.5 *	22.7 ± 0.4	19.5 ± 0.5 *	14.7 ± 0.2	11.5 ± 0.3 *	13.5 ± 0.2	12.0 ± 0.3 *
Magnesium (%)	15.7 ± 0.1	11.5 ± 0.1 *	30.3 ± 0.5	24.9 ± 0.8 *	24.5 ± 0.3	18.4 ± 0.4 *	21.2 ± 0.3	15.8 ± 0.4 *	13.6 ± 0.2	9.7 ± 0.3 *	12.3 ± 0.2	9.7 ± 0.2 *
MUFA (%)	14.0 ± 0.2	13.9 ± 0.2	25.9 ± 0.6	26.2 ± 0.9	18.4 ± 0.3	19.0 ± 0.5	15.9 ± 0.3	16.0 ± 0.4	13.2 ± 0.3	12.6 ± 0.4	11.8 ± 0.2	12.3 ± 0.3
Sodium (%)	13.8 ± 0.1	13.5 ± 0.2	21.9 ± 0.5	22.8 ± 0.8	17.1 ± 0.3	16.6 ± 0.4	15.4 ± 0.2	15.3 ± 0.3	13.3 ± 0.2	12.6 ± 0.3	11.8 ± 0.2	12.3 ± 0.3
Niacin (%)	11.6 ± 0.1	10.6 ± 0.2 *	22.2 ± 0.5	20.6 ± 0.6	16.9 ± 0.2	14.7 ± 0.3 *	14.8 ± 0.2	13.1 ± 0.3 *	10.4 ± 0.2	9.5 ± 0.3 *	9.5 ± 0.2	9.5 ± 0.2
Vitamin B6 (%)	9.8 ± 0.1	7.4 ± 0.1 *	18.5 ± 0.4	15.8 ± 0.6 *	14.4 ± 0.2	11.3 ± 0.3 *	12.8 ± 0.2	9.9 ± 0.3 *	8.7 ± 0.2	6.0 ± 0.2 *	7.9 ± 0.2	6.6 ± 0.2 *
Carbohydrates (%)	8.2 ± 0.1	7.7 ± 0.1	14.7 ± 0.4	15.8 ± 0.6	10.7 ± 0.2	11.2 ± 0.3	9.4 ± 0.2	9.1 ± 0.3	7.4 ± 0.2	6.5 ± 0.2 *	7.4 ± 0.1	7.3 ± 0.2
Thiamin (%)	8.0 ± 0.1	6.0 ± 0.1 *	16.2 ± 0.4	14.7 ± 0.6	11.1 ± 0.2	8.8 ± 0.3 *	9.7 ± 0.2	7.3 ± 0.2 *	7.1 ± 0.1	4.9 ± 0.2 *	6.8 ± 0.2	5.5 ± 0.1 *
Vitamin C (%)	5.4 ± 0.1	2.0 ± 0.1 *	7.3 ± 0.4	2.4 ± 0.4 *	6.8 ± 0.2	2.0 ± 0.2 *	7.2 ± 0.3	2.6 ± 0.3 *	5.1 ± 0.2	2.0 ± 0.2 *	4.6 ± 0.2	1.8 ± 0.1 *
Folate (%)	5.1 ± 0.1	4.5 ± 0.1 *	10.6 ± 0.3	9.4 ± 0.4	7.1 ± 0.1	6.0 ± 0.2 *	6.1 ± 0.1	5.3 ± 0.2 *	4.5 ± 0.1	3.8 ± 0.1 *	4.5 ± 0.1	4.3 ± 0.1
PUFA (%)	4.8 ± 0.1	4.8 ± 0.1	10.3 ± 0.4	10.6 ± 0.6	6.6 ± 0.2	6.4 ± 0.2	5.3 ± 0.1	5.5 ± 0.2	4.4 ± 0.1	4.3 ± 0.2	4.0 ± 0.1	4.2 ± 0.1
Iron (%)	3.0 ± 0.03	2.1 ± 0.04 *	5.4 ± 0.2	3.8 ± 0.2 *	3.9 ± 0.1	3.1 ± 0.1 *	3.6 ± 0.1	2.7 ± 0.1 *	2.8 ± 0.1	1.9 ± 0.1 *	2.3 ± 0.1	1.8 ± 0.1 *

All data are weighted and bootstrapped to obtain population level estimate. * Significant differences between 2004 and 2015 at 5% level of significance. ^#^The numbers (1 to 10) represent the top ten nutrients provided by Milk and Alternatives food group. Values are presented as % ± SE. CCHS: Canadian Community Health Survey; MUFA: Monounsaturated Fatty Acids; PUFA: Polyunsaturated Fatty Acids

As shown in Table 5, the percent contribution of energy from main food items of Milk & Alternatives food group remained relatively unchanged between 2004 and 2015, except for yogurt, which significantly decreased from 6.7 to 5.7%. In 2015, the top five nutrients provided by plain milk among plain milk consumers were vitamin D (49.3%), vitamin B12 (35.4%), calcium (33.4%), vitamin A (29.0%), and riboflavin (23.7%). Since the number of daily servings of plain milk among Canadians (≥ 2 years) significantly decreased from 2004 to 2015, plain milk provided lower proportions of the total intake of many nutrients in 2015 than in 2004 (Table 5). In 2015, the top five nutrients provided by flavoured milk among flavoured milk consumers were vitamin D (49.6%), calcium (33.6%), vitamin A (30.3%), vitamin B12 (28.6%), and total sugar (26.5%). In 2015, cheese contributed 29.1% of the calcium, 25.5% of the saturated fat, 20.2% of the vitamin A, 19.5% of the cholesterol, and 15.8% of the phosphorus in the diet of cheese consumers. In 2015, the top five nutrients provided by yogurt among yogurt consumers were calcium (18.3%), vitamin D (16.1%), vitamin B12 (13.7%), total sugar (13.7%), and riboflavin (11.8%). In addition, fortified soy beverages accounted for 37.0% of the vitamin D, 27.4% of the calcium, 25.2% of the vitamin B12, 16.0% of the vitamin A, and 15.9% of the riboflavin in the diet of fortified soy beverages consumers in 2015.

**Table 5:** Percent contribution of milk and milk alternatives to daily energy and nutrient intakes in 2004 and 2015^#^

	Plain milk		Flavoured milk		Cheese		Yogurt		Fortified soy beverages	
	CCHS 2004 (***n*** = 20,879,908)	CCHS 2015 (***n*** = 18,790,221)	CCHS 2004 (***n*** = 890,305)	CCHS 2015 (***n*** = 1,118,649)	CCHS 2004 (***n*** = 16,392,316)	CCHS 2015 (***n*** = 17,926,472)	CCHS 2004 (***n*** = 4,217,387)	CCHS 2015 (***n*** = 6,827,004)	CCHS 2004 (***n*** = 146,085)	CCHS 2015 (***n*** = 506,575)
Energy (%)	**7.8 ± 0.1**	**7.4 ± 0.1**	**12.2 ± 0.4**	**12.2 ± 0.4**	**8.0 ± 0.1**	**7.8 ± 0.1**	**6.7 ± 0.2**	5.7 ± 0.1 *	**4.0 ± 0.5**	**5.2 ± 0.4**
Vitamin D (%)	53.2 ± 0.4 ^1^	49.3 ± 0.5 ^1^*	49.9 ± 1.2 ^1^	49.6 ± 1.2 ^1^	4.9 ± 0.2	6.4 ± 0.3 *	2.6 ± 0.2	16.1 ± 0.6 ^2^*	43.0 ± 4.9 ^1^	37.0 ± 3.2 ^1^
Calcium (%)	36.8 ± 0.3 ^2^	33.4 ± 0.4 ^3^*	35.8 ± 1.0 ^2^	33.6 ± 1.0 ^2^	30.0 ± 0.3 ^1^	29.1 ± 0.4 ^1^	22.2 ± 0.5 ^1^	18.3 ± 0.4 ^1^*	27.6 ± 4.3 ^2^	27.4 ± 2.2 ^2^
Vitamin B12 (%)	32.6 ± 0.3 ^3^	35.4 ± 0.5 ^2^*	32.4 ± 1.1 ^3^	28.6 ± 1.0 ^4^	14.3 ± 0.2	14.8 ± 0.3	21.9 ± 0.6 ^2^	13.7 ± 0.4 ^3^*	25.0 ± 4.2 ^3^	25.2 ± 2.1 ^3^
Vitamin A (%)	28.5 ± 0.3 ^4^	29.0 ± 0.5 ^4^	31.4 ± 1.2 ^4^	30.3 ± 1.0 ^3^	17.7 ± 0.3 ^5^	20.2 ± 0.4 ^3^*	4.8 ± 0.3	5.2 ± 0.2	8.0 ± 1.2	16.0 ± 1.4 ^4^*
Riboflavin (%)	24.1 ± 0.2 ^5^	23.7 ± 0.3 ^5^	26.0 ± 0.8	25.2 ± 0.8	9.6 ± 0.1	9.4 ± 0.2	14.4 ± 0.3 ^3^	11.8 ± 0.3 ^5^*	14.7 ± 1.4	15.9 ± 1.2 ^5^
Phosphorus (%)	21.4 ± 0.2	18.6 ± 0.3*	24.9 ± 0.8	22.7 ± 0.7	18.4 ± 0.2 ^4^	15.8 ± 0.2 ^5^*	13.2 ± 0.3 ^5^	10.3 ± 0.2 *	8.0 ± 0.9	15.1 ± 1.2 *
Potassium (%)	16.4 ± 0.2	14.7 ± 0.2*	20.2 ± 0.6	19.5 ± 0.6	2.6 ± 0.03	2.8 ± 0.1	9.6 ± 0.2	7.8 ± 0.2 *	9.6 ± 1.0	10.5 ± 0.8
Total Sugar (%)	16.0 ± 0.2	15.8 ± 0.3	28.4 ± 1.1 ^5^	26.5 ± 0.9 ^5^	1.7 ± 0.1	1.5 ± 0.1*	13.3 ± 0.5 ^4^	13.7 ± 0.4 ^4^	1.4 ± 0.2	9.7 ± 0.8 *
Saturated Fat (%)	14.9 ± 0.2	14.5 ± 0.3	16.2 ± 0.7	13.4 ± 0.7*	27.7 ± 0.3 ^2^	25.5 ± 0.4 ^2^*	8.5 ± 0.5	7.2 ± 0.3	3.3 ± 0.5	2.9 ± 0.3
Magnesium (%)	14.0 ± 0.1	10.4 ± 0.2 *	16.3 ± 0.5	15.2 ± 0.6	4.5 ± 0.1	4.4 ± 0.1	7.8 ± 0.2	4.8 ± 0.1 *	11.5 ± 1.3	7.7 ± 0.7
Protein (%)	13.8 ± 0.1	12.5 ± 0.2 *	15.3 ± 0.5	13.6 ± 0.5	14.5 ± 0.2	13.0 ± 0.2*	8.4 ± 0.2	8.7 ± 0.2	7.1 ± 0.7	7.5 ± 0.5
Cholesterol (%)	13.3 ± 0.2	12.5 ± 0.3	12.1 ± 0.6	9.6 ± 0.7	19.5 ± 0.3 ^3^	19.5 ± 0.4 ^4^	7.3 ± 0.6	6.9 ± 0.3	**–**	**–**
Zinc (%)	12.6 ± 0.1	13.0 ± 0.2	15.0 ± 0.6	13.9 ± 0.5	13.0 ± 0.2	13.0 ± 0.2	10.0 ± 0.3	5.6 ± 0.1 *	8.0 ± 0.9	8.7 ± 0.7
Vitamin B6 (%)	8.7 ± 0.1	7.3 ± 0.1 *	10.3 ± 0.4	8.3 ± 0.4*	3.2 ± 0.1	2.6 ± 0.1*	4.7 ± 0.2	2.9 ± 0.1 *	4.7 ± 0.5	5.0 ± 0.4
Total Fat (%)	8.5 ± 0.1	8.3 ± 0.2	9.6 ± 0.5	7.7 ± 0.4*	16.3 ± 0.2	15.5 ± 0.3*	4.7 ± 0.3	4.0 ± 0.1	7.6 ± 1.2	5.6 ± 0.5
Niacin (%)	8.3 ± 0.1	6.7 ± 0.1 *	9.9 ± 0.4	8.0 ± 0.4*	7.4 ± 0.1	8.1 ± 0.2*	2.7 ± 0.1	3.9 ± 0.1 *	5.2 ± 0.6	7.1 ± 0.5
Thiamin (%)	7.9 ± 0.1	6.8 ± 0.1 *	9.3 ± 0.4	9.4 ± 0.5	1.0 ± 0.03	1.0 ± 0.03	4.8 ± 0.2	2.8 ± 0.1 *	17.3 ± 1.2 ^4^	5.5 ± 0.5*
Sodium (%)	6.3 ± 0.1	5.6 ± 0.1 *	8.6 ± 0.4	7.9 ± 0.3	12.9 ± 0.1	13.3 ± 0.2	4.0 ± 0.1	2.8 ± 0.1 *	1.3 ± 0.2	4.4 ± 0.4*
Carbohydrates (%)	6.3 ± 0.1	6.1 ± 0.1	14.1 ± 0.6	15.3 ± 0.5	0.8 ± 0.01	1.0 ± 0.03*	7.7 ± 0.2	6.4 ± 0.2 *	1.8 ± 0.3	4.7 ± 0.4*
MUFA (%)	6.2 ± 0.1	6.8 ± 0.1 *	7.7 ± 0.4	6.3 ± 0.4	12.7 ± 0.2	12.1 ± 0.2	3.8 ± 0.3	3.1 ± 0.1	3.8 ± 0.7	4.3 ± 0.4
Vitamin C (%)	5.8 ± 0.1	1.4 ± 0.1 *	6.1 ± 0.4	4.7 ± 0.5	**–**	**–**	3.6 ± 0.2	1.8 ± 0.2 *	**–**	3.7 ± 0.8
Folate (%)	4.4 ± 0.1	3.8 ± 0.1 *	5.2 ± 0.2	3.7 ± 0.2*	1.6 ± 0.03	2.2 ± 0.1*	3.7 ± 0.1	1.9 ± 0.1 *	1.4 ± 0.2	8.3 ± 0.6*
PUFA (%)	2.0 ± 0.03	2.2 ± 0.1	2.6 ± 0.1	1.8 ± 0.1*	3.7 ± 0.1	3.6 ± 0.1	1.0 ± 0.1	1.2 ± 0.1	15.2 ± 2.1 ^5^	13.4 ± 1.1
Iron (%)	1.3 ± 0.01	0.6 ± 0.02 *	7.2 ± 0.3	7.6 ± 0.4	2.3 ± 0.03	1.1 ± 0.02*	1.0 ± 0.04	1.4 ± 0.1 *	8.3 ± 0.7	7.2 ± 0.6

All data are weighted and bootstrapped to obtain population level estimate. * Significant differences between 2004 and 2015 at 5% level of significance. ^#^ The numbers (1 to 5) represent the top five nutrients provided by milk and milk alternatives. Values are presented as % ± SE. CCHS: Canadian Community Health Survey; MUFA: Monounsaturated Fatty Acids; PUFA: Polyunsaturated Fatty Acids

## Discussion

In the present study, using nationally representative samples, we aimed to report changes in intake of Milk & Alternatives in the Canadian population (≥ 2 years) from 2004 to 2015. Overall, both the proportion of Canadians consuming Milk & Alternatives and the number of servings consumed per day significantly decreased from 2004 to 2015. Prevalence of meeting recommendations for Milk & Alternatives food group among Milk & Alternatives consumers significantly decreased from 28.3% in 2004 to 24.4% in 2015. Milk & Alternatives were major contributors to calcium, vitamin D, and vitamin B12 intakes in the diet of Canadians in both time points. Milk & Alternatives consumers obtained about 68 and 56% more saturated fat than non-consumers in 2004 and 2015, respectively.

From 2004 to 2015, the proportion of Canadians consuming Milk & Alternatives significantly decreased from 89.5 to 87.7% and the number of servings consumed per day dropped from 1.9 to 1.7. According to the data provided by the USDA, the per capita dairy product availability in the United States increased about 9%, from 595 pounds in 2000 to 646 pounds in 2018 [5]. This increase was mainly due to an increase in cheese consumption. However, the per capita consumption of fluid milk including whole milk reduced-fat milk, skim milk, flavoured milk, buttermilk, and other types of milk decreased about 26%, from 197 pounds in 2000 to 146 pounds in 2018 [5]. Using two cross-sectional national surveys conducted in France (e’tude Individuelle Nationale sur les Consommations Alimentaires), Lioret et al. found that the overall intake of dairy products decreased by 10 and 12%, respectively, in French children aged 3–10 years and 11–14 years between 1999 and 2007. This downward trend in consumption of dairy products was mainly due to the marked decrease in consumption of milk [19]. Further, our results revealed that Immigrants to Canada were more likely to be Milk & Alternatives non-consumers. The sociodemographics of immigrants is changing over time and culture is playing an important role in food preference. Further investigation is required to look into dietary laws and cultural preferences of the Immigrant population in Canada.

The decrease in consumption of Milk & Alternatives between 2004 and 2015 mainly resulted from significant decreases in the percent of plain milk consumers (from 70.2 to 56.1%) and the number of daily servings of plain milk (from 1.2 to 0.9 cups) and flavoured milk (from 1.49 to 1.29 cups). Recent evidence suggests that there is a general tendency toward decreasing the consumption of milk in developed countries [5–7]. Using data from National Health and Nutrition Examination Survey (NHANES), Bleich et al. reported that the percentage of US adults (≥20 years) consuming milk decreased from 44.2–54.9% in 2003–04 to 35.2–44.8% in 2013–14. During this time period, the per capita consumption of milk calorie among US adults also decreased from 73 to 102 kcal to 62–68 kcal [20]. The percentage of US adolescents (12–19 years) consuming milk and the per capita consumption of milk calories among adolescents also decreased from 56.3 to 51.7% and 145 kcal to 105 kcal, respectively, between 2003 and 04 and 2013–14 [20]. Further, according to a report by the USDA, Americans aged 13 and over tended to drink 0.8 cups of fluid milk per day in 1977–78, which decreased to 0.6 cups per day in 2007–08 [21].

Our results revealed that between 2004 and 2015, the proportion of Canadian children aged 2–5 years and 6–12 years who reported consuming plain milk decreased from 87.4 to 81.3% and from 81.4 to 71.2%, respectively. The proportion of Canadian children aged 6–12 years who reported consuming flavoured milk increased from 7.5% in 2004 to 11.1% in 2015. Consistent with our findings, Bleich et al. reported that from 2003 to 04 to 2013–14 the percentage of US children aged 2–5 years and 6–11 years consuming milk decreased from 84.6 to 78.4% and from 76.4 to 72.6%, respectively [20]. Between 2003 and 04 and 2013–14, the per capita consumption of milk calories among US children aged 2–5 years and 6–11 years also decreased from 230 kcal to 157 kcal and from 202 kcal to 140 kcal, respectively [20]. Further, fluid milk intake in preadolescent children decreased from 1.7 cups per day in 1977–1978 to 1.2 cups per day in 2007–2008 [22]. Using data from three US nationally representative dietary recall surveys, Lasater et al. found that consumption of flavoured milk containing added sugar (high fat plus low fat) among US children increased significantly from 29 kcal/day to 68 kcal/day [21]. Similarly, the percentage of US children < 1–5 years of age consuming flavoured milk was relatively low during 1976–1980 and 1988–1994, but increased to 14% during the NHANES 2001–2006 [23].

From 2004 to 2015, the proportion of Canadians consuming cheese remained relatively unchanged (55.1% vs 53.5%), but the number of daily servings of cheese rose significantly from 0.7 to 0.8. In addition, the proportion of Canadians consuming yogurt increased significantly from 14.2% in 2004 to 20.4% in 2015 but the number of daily servings did not change significantly. The global cheese consumption increased at a rate of ~ 1.5% per annum between 1990 and 2000 and 2.5% between 2000 and 2007 [24] but remained relatively unchanged between 2007 and 2013 [25]. In the United States, cheese and yogurt contributed significantly to the overall increase in dairy product availability over time [5]. The per capita consumption of cheese (other than cottage cheese) in the US population increased from 29.6 pounds per person in 2000 to 34.2 pounds per person in 2018. During this time period, the per capita consumption of yogurt increased from 6.5 pounds per person to 13.4 pounds per person [5]. Per capita cheese consumption across the European Union increased from 16.7 kg/person/annum in 2007 [26] to 17.9 kg/person/annum in 2014 [27]. In Finland, the consumption of cheese more than doubled during the last few decades and the consumption of yogurt showed an upward trend [6]. The per capita consumption of yogurt has increased in recent decades because many consumers associate yogurt with good health [28].

From 2004 to 2015, the prevalence of meeting recommendations for Milk & Alternatives food group among Milk & Alternatives consumers significantly decreased from 28.3 to 24.4%. In a study conducted in a Canadian university community (653 staff members, 2490 students), more than 49% of staff members and about 46% of students did not meet the recommendations for Milk & Alternatives [29]. Kolahdooz et al. conducted a study in a sample of Alberta’s multi-ethnic youths aged 11–23 years and found that the percentage of youths not meeting recommendations for Milk & Alternatives was 81.7% for Indigenous, 73.3% for African & Middle Eastern, 78.6% for Asian and 63.5% for European youths [30]. Results from a longitudinal study conducted in a sample of Canadian grade 9–12 students (ages 13–18 years) showed that only a quarter of students (24.9%) met the recommendations for Milk & Alternatives at both time points [31]. These results clearly suggest that the majority of Canadians do not consume minimum daily recommended servings of Milk & Alternatives.

Despite their low energy contribution (12.3% of energy), Milk & Alternatives contributed 45.8% of calcium, 39.9% of vitamin D, and 36.0% of vitamin B12 to the diet of the Canadian population in 2015. Milk & Alternatives were among the top sources of vitamin A, phosphorus and riboflavin. Using data from the 4 cycles of continuous NHANES 2001–2002, 2003–2004, 2005–2006, and 2007–2008, Drewnowski showed that milk and milk products contributed 10–13% of total energy intake, 47% of calcium intake, 42% of retinol intake and 65% of vitamin D intake in US children and adults. Milk and milk products were also major sources of riboflavin, phosphorus, and vitamin B12 in the US diet [32]. A nationwide cross-sectional survey (2008–09) conducted in the Brazilian population aged ≥10 years showed that dairy products accounted for 6.1% of daily energy, 37.9% of calcium, 35.9 of vitamin D, 18.7% of riboflavin, and 17.0% of phosphorus [33]. Vissers et al. assessed the contribution of dairy products to the intake of micronutrients in the Dutch population. Dairy products contributed 73% of calcium, 58% of vitamin B12, and 39% of zinc in young Dutch children. In adults and elderly subjects, dairy products contributed 65–68% of calcium, 44–46% of vitamin B12 and 28–31% of zinc [34]. Dairy products were the top sources of calcium (45.6%), iodine (29.8%) and riboflavin (28.2%), and the second most important source of phosphorus (24.7%), zinc (19.6%), retinol (16.5%), vitamin B12 (14.5%), and vitamin D (14.3%) in French adults (18–79 years). Moreover, dairy products were the first source of calcium (53.2%), iodine (39.7%), riboflavin (38.4%), phosphorus (31%), and potassium (21.4%), and the second major source of zinc (24.7%), vitamins B5 (24.6%), retinol (24.1%), vitamin B12 (22.6%), and vitamin D (18.7%) in French children (3–17 years) [35].

We showed that Milk & Alternatives food group was a significant contributor to total fat and saturated fat intakes in the Canadian diet (18.3 and 31.2%, respectively, in 2004 and 17.3 and 28.6%, respectively in 2015). Further, Milk & Alternatives consumers obtained about 68 and 56% more saturated fat than non-consumers in 2004 and 2015, respectively. Milk & Alternatives, especially cheese, has been recognized as major sources of saturated fat in the diet of most populations [33, 36–39]. Using nationally representative data from the National Adult Nutrition Survey (NANS), Feeney et al. reported that dairy products and cheese contributed to near 20 and 8.3% of saturated fat intake in the Irish population, respectively [36]. Data from the 2008–2009 Brazilian Household Budget Survey showed that dairy products accounted for 10.0% of total fat and 16.9% of saturated fat intake in the Brazilian population aged ≥10 years [33]. Using data from the Western Australian Pregnancy Cohort (Raine) Study, O’Sullivan et al. reported that dairy products (excluding butter) contributed near 21% of total fat and 31.5% of saturated fat intake in Australian adolescents aged 13–15 years [37]. Dairy products (excluding butter) accounted for 24 and 39%, respectively, of total fat and saturated fat intakes in US children and adolescents aged 2–18 years [38], and 20% of total fat and 34.6% of saturated fat intake in the Spanish population aged 2 to 24 years [39]. The high contribution of dairy products to saturated fat intake together with the undesirable effects of saturated fat consumption on human health [40–42] may explain why people generally tend to decrease their dairy food intake. Further, in efforts to decrease saturated fat consumption, the new 2019 Canada’s Food Guide has eliminated the “Milk & Alternatives” food category and recommended only lower-fat dairy products as a subgroup of protein foods [4]. However, this may accelerate the previously observed downward trend in dietary intakes of calcium and vitamin D [43, 44], and alter the nutritional profile of the diet of Canadians.

### Study limitations

This study used nutrition data from two nationally representative surveys of the Canadian population, CCHS 2004 (Cycle 2.2) and 2015 CCHS-Nutrition. A major strength of this study was the opportunity to examine the changes in Milk & Alternatives consumption among Canadians, shortly after the introduction of new Canada’s food guide. We also acknowledge some limitations. First, changes to the food booklet have resulted in some portion size changes, especially in the case of beverages. For example, changes to the food portion size booklet resulted in a decrease of 39 mL (429 mL in 2004 vs 390 mL in 2015) and 55 mL (325 mL in 2004 vs 270 mL in 2015) in quantities of the 2 largest drinking glass options [45]. Thus, caution should be taken when comparing the results of two surveys. Second, because the 2004 and 2015 data were analyzed separately, the comparisons were made by a 95% CI overlapping method. Third, data regarding Milk & Alternatives consumption was obtained using a 24-h recall, which is a self-report method subject to misreporting (i.e., overestimating or underestimating dietary intake). Fourth, our results indicate statistical significant differences, whether such differences are meaningful from clinical and public health perspectives warrants further investigation.

### Study implications and usefulness

This study showed that there has been a decreasing trend in consumption of Milk & Alternatives in Canada from 2004 to 2015. Transition of Milk & Alternatives from one of the four food groups of 2007 Canada’s food guide to only a source of protein in the new Canada’s Food Guide (2019) may further exacerbate the observed downward trend in consumption of dairy products. Further, this decreasing trend may decrease the dietary intake of some major nutrients, in particular, calcium and vitamin D, and adversely affect the nutritional profile of the diet. In this regard, a recent study reported that the current Canada’s Food Guide Snapshot appears to be nutritionally inadequate in terms of calcium and vitamin D for Canadian youth [46]. Therefore, in the face of new recommendations, policy-makers, stakeholders and consumers must take caution to ensure adequate intake of nutrients. The new Canada’s Food Guide has also recommended consuming fortified-soy beverages as a plant-based source of protein [4]. Consumption of these plant-based milk alternatives can be considered as an option to prevent nutrient deficiencies in Milk & Alternatives non-consumers. This research serves as a baseline with which to compare future surveys on consumption of Milk & Alternatives, in the light of the recommendations of new Canada’s Food Guide.

## Conclusion

In conclusion, we used 2 nationally representative surveys to examine changes in intake of Milk & Alternatives among Canadians over an 11-year period. Both the proportion of Canadians consuming Milk & Alternatives and the number of servings consumed per day significantly decreased from 2004 to 2015. This downward trend in consumption of Milk & Alternatives resulted in a significant decrease in meeting Canada’s Food Guide (2007) recommendations for Milk & Alternatives. Despite their low energy contribution, Milk & Alternatives were among the top sources of calcium, vitamin D, vitamin B12, vitamin A, phosphorus and riboflavin. Milk & Alternatives food group was a major contributor to total fat and saturated fat intake in the Canadian diet.

## Data Availability

The data used in this study is available at Statistics Canada Research Data Centres (RDCs). RDCs are secure physical environments available to accredited researchers and government employees to access anonymized microdata for research purposes. RDCs are located on university campuses across Canada and are staffed by Statistics Canada employees. Accessing data requires specific permission by Statistics Canada. More detailed information can be found via https://www.statcan.gc.ca/eng/microdata

## Appendix

**Table 6:** Description of Milk and alternatives food group and the main food items

Food group/subgroup	Food codes description	Food content
Milk and alternatives	CFG	Fluid milk and fortified soy-based beverages and other milk alternatives such as cheese and yogurt
Fluid milk	CNF	Skim, 1, 2, 3.25% or whole milk
Flavoured milk	CNF	All types of flavoured milk, e.g. strawberry, vanilla, chocolate etc.
Cheese	CNF	All kinds of cheese products including cheddar, blue, cream, parmesan, cheese spread etc.
Yogurt	CNF	Skim 1, 2, 3.25% or full fat yogurt and flavoured yogurt
Fortified soy beverages	CNF	All fortified soy beverages; does not include plant based beverages such as coconut, almond, cashew milk

## Abbreviations

PUFA: polyunsaturated fatty acids
MUFA: monounsaturated fatty acids
SFA: saturated fatty acids
CCHS: Canadian Community Health Survey
BMI: body mass index
NDNS: National Diet and Nutrition Survey
AMPM: Automated Multiple Pass Method
WHO: World Health Organization
NHANES: National Health and Nutrition Examination Survey
